# Multifocal xanthogranulomatous inflammation of the urinary tract mimicking metastatic urothelial carcinoma: A case report

**DOI:** 10.1097/MD.0000000000049724

**Published:** 2026-07-17

**Authors:** Zhongwei Zhang, Yuwan Zhao, Zhuo Li

**Affiliations:** a Laboratory of Urology, Affiliated Hospital of Guangdong Medical University, Zhanjiang, Guangdong, China; b Department of Urology, Luchuan County People’s Hospital, Luchuan, Guangxi, China.

**Keywords:** case report, diabetic immunocompromise, multifocal urinary tract disease, xanthogranulomatous inflammation

## Abstract

**Rationale:**

Xanthogranulomatous inflammation (XGI) is a rare chronic granulomatous disorder characterized by lipid-laden macrophage infiltration and most commonly involving the kidney and gallbladder. Multifocal urinary tract involvement is exceptionally rare and may radiologically mimic metastatic malignancy, particularly on positron emission tomography-computed tomography (PET/CT).

**Patient concerns:**

A 67-year-old woman with long-standing diabetes mellitus and hypertension presented with left hydronephrosis, recurrent urinary tract infections, and progressive renal dysfunction. PET/CT demonstrated extensive hypermetabolic lesions involving the bladder, ureters, renal pelvis, and retroperitoneal lymph nodes, raising suspicion for metastatic urothelial carcinoma.

**Diagnoses:**

Multifocal xanthogranulomatous inflammation involving the bladder and ureter, with radiologically suspected upper urinary tract involvement.

**Interventions:**

The patient underwent transurethral resection of the left ureteral orifice and bladder lesions, bilateral nephrostomy, renal abscess drainage, and antibiotic therapy.

**Outcomes:**

Repeated histopathological and immunohistochemical examinations consistently excluded malignancy and confirmed xanthogranulomatous inflammation. Following surgical drainage and anti-infective treatment, infection parameters improved substantially, and the patient was discharged in stable condition.

**Lessons:**

PET/CT hypermetabolism does not exclude inflammatory disease. When imaging findings strongly suggest malignancy, repeated histopathological evaluation remains essential for establishing an accurate diagnosis. Multifocal urinary tract XGI should be considered in the differential diagnosis of PET-positive urinary tract lesions, particularly in patients with diabetes and recurrent urinary tract infections.

## 1. Background

Xanthogranulomatous inflammation (XGI) is a rare chronic granulomatous disorder characterized by lipid-laden macrophage infiltration, typically occurring as a localized process in organs such as the kidney (xanthogranulomatous pyelonephritis, XGP) or gallbladder.^[1,2]^ While unilateral renal XGP is well-documented, multifocal involvement beyond a single organ is exceedingly uncommon. Furthermore, multifocal urinary tract XGI involving multiple anatomical sites has only rarely been described in the literature.^[3]^

Diagnostic challenges arise from its radiological tendency to mimic malignancy, particularly on FDG-PET/CT, where hypermetabolic lesions may falsely suggest metastatic urothelial carcinoma.^[4]^ This discordance underscores the critical role of histopathological confirmation. Additionally, although immunocompromised states, such as uncontrolled diabetes, are recognized risk factors due to impaired bacterial clearance and dysregulated macrophage responses, the precise immunometabolic mechanisms potentially contributing to extensive xanthogranulomatous inflammation remain poorly understood.

We present a case of pathologically confirmed xanthogranulomatous inflammation involving the bladder and ureteral orifice, accompanied by radiologically extensive upper urinary tract and retroperitoneal abnormalities, in a diabetic patient whose condition was initially misdiagnosed as metastatic cancer by PET/CT. This case aims to augment the sparse literature on multifocal urinary tract XGI and refine diagnostic strategies for this masquerading entity.

## 2. Caset presentation

A 67-year-old female with poorly controlled diabetes (HbA1c 9.2%) and an 8-year history of hypertension presented to our hospital in February 2023 with left hydronephrosis. The patient underwent transurethral resection of the left ureteral orifice with electrocautery of the bladder mucosa. Histopathological examination revealed xanthogranulomatous inflammation at the left ureteral orifice (Fig. 1), Immunohistochemical staining demonstrated the following profile: CK(−) (excluding epithelial origin), CD68(+) (confirming the presence of histiocytes), Ki-67(10%) (indicating low proliferative activity), S-100(−), CD1a(−), and CD163(−) [excluding histiocytic disorders such as Rosai–Dorfman and Erdheim–Chester diseases (ECDs)], CD3(+) (background T cells), CD20(+) (background B cells), and ALK(5A4)(−) (excluding inflammatory myofibroblastic tumor). In situ hybridization (EBER Probe) and special stains (PAS, Grocott methenamine silver; excluding fungal infections) were negative. The patient was discharged after her condition stabilized.

**Figure 1.**
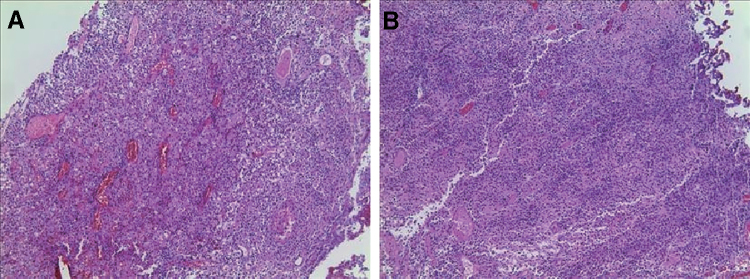
Histopathology of the left ureteral orifice (A) H&E staining (×100) shows mucosal erosion with dense infiltration of lipid-laden macrophages (B) CD68 IHC (×400) confirms abundant foamy histiocytes, characteristic of xanthogranulomatous inflammation. H&E = hematoxylin and eosin, IHC = immunohistochemistry.

On September 23, 2024, the patient was readmitted to Nanfang Hospital with elevated serum creatinine persisting for 1 month. Laboratory tests showed renal dysfunction (creatinine 229 μmol/L, urea 13.6 mmol/L, eGFR 18.43 mL/min). Cystoscopy revealed multiple masses in the trigone, left wall, right wall, and posterior wall of the bladder. Transurethral resection of bladder lesions was performed on September 24, followed by epirubicin bladder perfusion. Renal function progressively deteriorated (creatinine 453 μmol/L, eGFR 12.25 mL/min by September 29), prompting percutaneous bilateral nephrostomy.

PET-CT on September 30 demonstrated hypermetabolic lesions in the bladder, bilateral ureters, renal pelvis, cervix, and lymph nodes (SUVmax 8.2; Fig. 2), initially suggestive of urothelial malignancy. Subsequently, cervical liquid-based cytology (TCT) was performed, revealing a high-grade squamous intraepithelial lesion (HSIL). This was accompanied by cell DNA quantification showing a large number of DNA aneuploid cells. Therefore, the cervical hypermetabolism was confirmed as a concurrent, independent gynecological lesion rather than the dissemination of XGI. Meanwhile, histopathological findings from the bladder biopsy on October 10 confirmed chronic inflammation with xanthogranuloma formation (Fig. 3). Immunohistochemistry showed: CK(+)(highlighting reactive epithelium), CD68(+)(confirming histiocytes), Ki-67 (15%), with negative staining for CK20(excluding urothelial carcinoma) and B-RAF-V600E(further excluding Erdheim–Chester disease). Special stains for fungi were negative. The patient was treated with piperacillin-tazobactam and discharged in stable condition.

**Figure 2.**
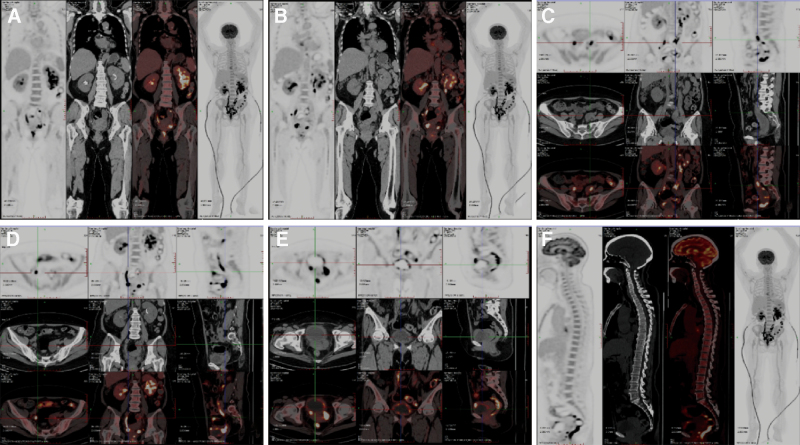
PET-CT findings during disease progression (September 2024). (A) Bilateral renal pelvis soft-tissue filling with hypermetabolism (SUVmax 8.7). (B) Renal cortical hypermetabolism with fascial thickening, suggesting pyelitis. (C) Focal hypermetabolic thickening of left ureter. (D) Hypermetabolic lesion at right distal ureter. (E) Heterogeneous bladder wall thickening with hypermetabolism. (F) Nodular hypermetabolic lesions in cervix. PET-CT = positron emission tomography-computed tomography, SUVmax = maximum standardized uptake value.

**Figure 3.**
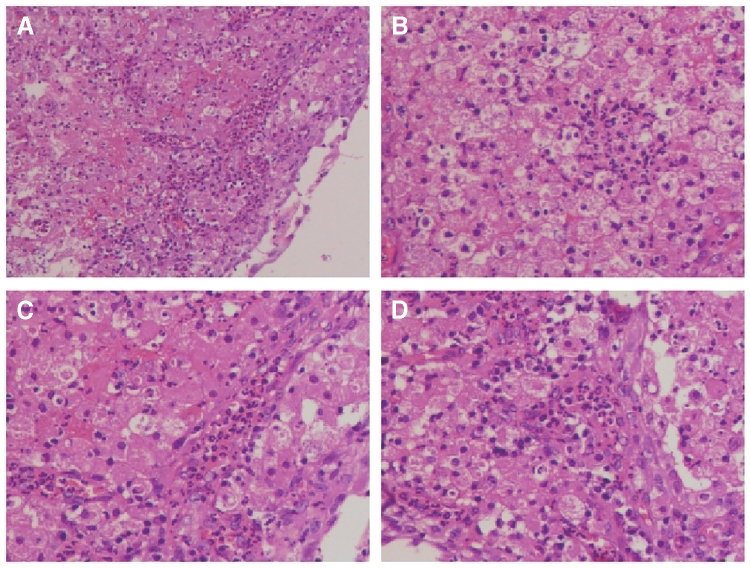
Histopathology of bladder lesions (October 2024). (A–D) H&E staining (×200) reveals chronic inflammation with extensive xanthogranuloma formation across different microscopic fields. Dense infiltrates of lipid-laden macrophages (foamy histiocytes) are visible, with no morphological evidence of cellular atypia or malignancy. H&E = hematoxylin and eosin.

The patient was readmitted on January 3, 2025 with fever and left flank pain 3 months post-nephrostomy. Laboratory tests revealed urinary tract infection (WBC 54,000/μL, pyuria 2+/HPF), anemia (Hb 51 g/L), and elevated inflammatory markers (CRP 153 mg/L, PCT 30.2 ng/mL). CT urography (January 6) showed left renal abscess, diffuse ureteral wall thickening, and retroperitoneal lymphadenopathy (Fig. 4). Following abscess drainage on January 9 and meropenem therapy, infection parameters improved (WBC 11.5 × 10^9^/L, PCT 0.217 ng/mL by January 24), though anemia persisted (Hb 66 g/L post-transfusion).

**Figure 4.**
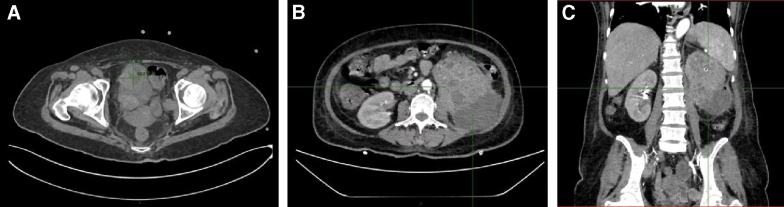
CT urography during renal abscess progression (January 2025). (A) Axial contrast-enhanced CT: Heterogeneous bladder wall thickening with nodular enhancement (arrows) and involvement of the anterior uterine wall (B) and (C) coronal reconstruction: Left renal enlargement with multiple non-enhancing hypodense areas indicating abscesses, loss of pelvicalyceal architecture, and retroperitoneal lymphadenopathy. CT = computed tomography.

## 3. Discussion

This case of multifocal urinary tract xanthogranulomatous inflammation (XGI) underscores several critical challenges in diagnosing and managing this rare entity. Histopathological confirmation of xanthogranulomatous inflammation was limited to the bladder and left ureteral orifice lesions, whereas the upper urinary tract and retroperitoneal abnormalities were assessed primarily by radiologic imaging. This represents an exceptionally rare manifestation of XGI, which is typically confined to a single organ (e.g., xanthogranulomatous pyelonephritis). The clinical and radiological mimicry of metastatic malignancy, coupled with histopathological ambiguity, highlights the need for a multidisciplinary diagnostic approach and raises questions about the possible association between chronic inflammation and metabolic dysfunction in XGI.

### 3.1. Radiological-pathological discordance: malignancy mimicry

The initial PET-CT findings met PERCIST criteria for malignancy,^[5]^ with hypermetabolic lesions (SUVmax 8.7 in the bladder, 7.8 in retroperitoneal nodes) and an anatomical distribution suggestive of urothelial carcinoma with metastatic spread. However, repeated histopathological examinations consistently demonstrated chronic inflammation with xanthogranuloma formation, without evidence of malignancy on repeated histopathological examinations.^[6]^ This discrepancy may be attributed to the following mechanisms:

**A**. Para-inflammatory lymphatic activation: Chronic granulomatous inflammation can induce reactive lymph node hyperplasia with increased glucose metabolism,^[7]^ mimicking metastatic spread. Similar phenomena have been reported in IgG4-related diseases and sarcoidosis.

**B.** Macrophage-driven FDG avidity**: Lipid-laden CD68+/Lys + macrophages, the hallmark of XGI, exhibit upregulated glycolytic activity, contributing to false-positive PET findings.^[8]^

**C.** Tumor-like angiogenesis**: Progressive tissue destruction and reparative neovascularization in chronic inflammation may enhance radiotracer uptake, as seen in this patient’s bladder and ureteral lesions^[9]^

This case emphasizes the necessity of histopathological confirmation for PET-positive lesions, even when clinical and imaging features strongly suggest malignancy.

### 3.2. Possible association between diabetes and chronic inflammation

The patient had poorly controlled diabetes mellitus (HbA1c 9.2%), which may have contributed to impaired immune responses and persistent urinary tract inflammation. Previous studies have suggested that chronic hyperglycemia may influence macrophage activity, bacterial clearance, and inflammatory signaling pathways.^[10,11]^ However, the precise relationship between diabetes and diffuse xanthogranulomatous inflammation remains unclear and was not directly investigated in this case. Therefore, the observations in this report should be interpreted cautiously and regarded as hypothesis-generating rather than evidence of a definitive pathogenic mechanism.

### 3.3. Differential diagnosis

Given the extensive distribution of hypermetabolic lesions on ^18^FDG PET/CT, a rigorous differential diagnosis is paramount to distinguish xanthogranulomatous inflammation (XGI) from other chronic granulomatous disorders, histiocytic proliferation diseases, and occult malignancies. Therefore, 7 key entities were systematically evaluated and excluded based on comprehensive histopathological, immunohistochemical, and laboratory findings:

#### 3.3.1. Malakoplakia

Structurally, malakoplakia closely mirrors XGI due to dense histiocytic infiltration. However, it is pathognomonically characterized by Michaelis–Gutmann (M–G) bodies – calcified, concentric, basophilic cytoplasmic inclusions that typically exhibit strong periodic acid-Schiff (PAS) and Von Kossa positivity. In our patient, although immunohistochemistry confirmed abundant CD68^+^ and Lys^+^ foamy histiocytes, the special staining for PAS was uniformly negative, and no M–G bodies were identified under light microscopy, effectively excluding malakoplakia.

#### 3.3.2. IgG4-related disease (IgG4-RD)

IgG4-RD can involve the retroperitoneum and urinary tract, manifesting as diffuse wall thickening or pseudo-tumoral masses accompanied by dense lymphoplasmacytic infiltrates. While specific serum IgG4 levels were not directly measured, the diagnosis of IgG4-RD typically requires characteristic histopathological features, notably a dense lymphoplasmacytic infiltrate, storiform fibrosis, and obliterative phlebitis, which were entirely absent in our patient’s biopsies. Furthermore, the patient’s serum immunoglobulin panel (IgG, IgA and IgM) and serum protein electrophoresis were within normal limits without evidence of hypergammaglobulinemia, thereby clinically and pathologically ruling out an active IgG4-mediated process.

#### 3.3.3. Genitourinary tuberculosis

Chronic granulomatous inflammation driven by *Mycobacterium tuberculosis* typically presents with caseating necrosis and Langhans giant cells. In the present case, histopathological examination revealed no caseating necrosis. Furthermore, peripheral blood tuberculosis IgG antibody and plasma TB-DNA PCR testing were both negative, and multiple acid-fast staining (AAS) series performed on the bladder and ureteral orifice tissues were consistently negative, ruling out tuberculosis.

#### 3.3.4. Chronic fungal infection

Systemic or localized opportunistic fungal infections can induce chronic granulomatous reactions, particularly in immunocompromised individuals such as patients with poorly controlled diabetes mellitus. Despite our patient’s high glycated hemoglobin level (HbA1c 9.2%), serological testing for specific fungal antibodies (Aspergillus fumigatus and Alternaria alternata) and serum (1,3)-β-D-glucan (G-test) levels (<10, negative) returned completely negative results. Furthermore, multiple specialized mycological investigations – including Grocott methenamine silver staining, PAS staining, fungal immunofluorescence, and midstream urine fungal cultures – were all negative, firmly eliminating a fungal etiology.

#### 3.3.5. Erdheim–Chester disease and Rosai–Dorfman disease

These non-Langerhans cell histiocytoses frequently present with retroperitoneal fibrosis or multi-organ involvement. Rosai–Dorfman Disease histiocytes are typically characterized by strong S-100 positivity and emperipolesis, while ECD features lipid-laden histiocytic infiltration with CD68^+^/ CD163^+^ profiles and a high frequency of the B-RAF-V600E mutation. In our patient, immunohistochemical analysis demonstrated that the infiltrating histiocytes were negative for B-RAF-V600E and CD163, with only scattered, negligible expression of S-100, and a total absence of emperipolesis, thus eliminating both histiocytic disorders.

#### 3.3.6. Urothelial carcinoma with secondary inflammatory reaction

High-grade urothelial carcinoma can elicit a profound granulomatous or xanthogranulomatous stromal response, which may mask malignant cells on small biopsies and generate false-positive PET/CT findings. In this case, urothelial malignancy of the urinary tract was rigorously excluded by consecutive negative results of urine liquid-based cytology for 3 days, the absence of malignant features in repeated tissue biopsies, and negative immunohistochemical staining for CK20 in the bladder lesions.

Importantly, the hypermetabolic cervical lesion (SUVmax 8.2), initially suspected to be a metastatic site, was conclusively diagnosed via cervical liquid-based cytology (TCT) as a coexisting high-grade squamous intraepithelial lesion (HSIL). This was further substantiated by cellular DNA quantification revealing a large population of DNA aneuploid cells. This critical finding untangles the diagnostic dilemma, demonstrating that the cervical hypermetabolism represents a concurrent, independent gynecological pathology rather than the anatomical extension of multifocal urinary tract XGI or urothelial carcinoma metastasis. The chronic inflammatory process in the urinary tract was driven by a verified *Escherichia coli* infection, as captured in midstream urine cultures, which aligns with the classic infectious etiology of XGI.

## 4. Conclusion

Multifocal xanthogranulomatous inflammation of the urinary tract is a rare inflammatory condition that may mimic metastatic malignancy, presenting significant challenges in diagnosis and treatment. This case suggests 2 key lessons: 1. PET-CT hypermetabolism does not rule out inflammatory disease.2. When imaging examination indicates malignancy, repeated histopathological examination must be performed to confirm the diagnosis. Clinicians should improve their awareness of the disease by combining clinical manifestations, imaging findings, and pathological examinations to establish a clear diagnosis as soon as possible, and develop an individualized treatment plan according to the specific condition of the patient. For diabetic patients with multifocal urinary tract lesions showing PET hypermetabolism, we recommend: tissue biopsy prior to anticancer therapy; strict glycemic control as an adjuvant anti-inflammatory strategy. As the disease is rare, the understanding of the disease needs to be improved, and it is necessary to conduct more case reports and studies to enhance diagnosis and treatment protocols.

## Author contributions

**Investigation:** Zhongwei Zhang.

**Project administration:** Zhuo Li.

**Supervision:** Zhuo Li.

**Writing – original draft:** Zhongwei Zhang, Yuwan Zhao.

**Writing – review & editing:** Zhuo Li.

## References

[1] Craig WD Wagner BJ Travis MD. Pyelonephritis: radiologic-pathologic review. Radiographics. 2008;28:255–77; quiz 327. 18203942 10.1148/rg.281075171

[2] Korkes F Favoretto RL Bróglio M Silva CA Castro MG Perez MD. Xanthogranulomatous pyelonephritis: clinical experience with 41 cases. Urology. 2008;71:178–80. 18308077 10.1016/j.urology.2007.09.026

[3] Cheng TY Tang TY Hsu JM Lin TF. Multifocal Xanthogranulomatous cystitis mimicking bladder malignancy: A case report and literature review. Asian J Surg. 2023;46:969–70. 35961900 10.1016/j.asjsur.2022.07.080

[4] Cheng G Torigian DA Alavi A. FDG PET/CT and MRI findings in a patient with focal xanthogranulomatous pyelonephritis mimicking cystic renal malignancy. Clin Nephrol. 2011;76:484–6. 22105452 10.5414/cn106762

[5] Wahl RL Jacene H Kasamon Y Lodge MA. From RECIST to PERCIST: evolving considerations for PET response criteria in solid tumors. J Nucl Med. 2009;50(Suppl 1):122S–50S. 19403881 10.2967/jnumed.108.057307 PMC2755245

[6] Wang Y Han XC Zheng LQ Miao WL. Xanthogranulomatous cystitis imitating bladder neoplasm: a case report and review of literature. Int J Clin Exp Pathol. 2014;7:8255–8. 25550882 PMC4270612

[7] Garg G DaSilva R Bhalakia A Milstein DM. Utility of fluorine-18-fluorodeoxyglucose positron emission tomography/computed tomography in a child with chronic granulomatous disease. Indian J Nucl Med. 2016;31:62–4. 26917900 10.4103/0972-3919.172366 PMC4746847

[8] Glaudemans AW de Vries EF Galli F Dierckx RAJO Slart RHJA Signore A. The use of (18)F-FDG-PET/CT for diagnosis and treatment monitoring of inflammatory and infectious diseases. Clin Dev Immunol. 2013;2013:623036. 24027590 10.1155/2013/623036 PMC3763592

[9] Jamar F Buscombe J Chiti A. EANM/SNMMI guideline for 18F-FDG use in inflammation and infection. J Nucl Med. 2013;54:647–58. 23359660 10.2967/jnumed.112.112524

[10] Hotamisligil GS. Inflammation and metabolic disorders. Nature. 2006;444:860–7. 17167474 10.1038/nature05485

[11] Confederat LG Condurache MI Alexa RE Dragostin OM. Particularities of urinary tract infections in diabetic patients: a concise review. Medicina (Kaunas). 2023;59:1747. 37893465 10.3390/medicina59101747 PMC10608443

